# Modified unilateral iliac screw fixation with partial reduction in the treatment of high-grade spondylolisthesis at L5/S1 in adult patients: introduction of key technique, report of clinical outcomes and analysis of spinopelvic parameters

**DOI:** 10.1186/s12891-023-06552-1

**Published:** 2023-06-02

**Authors:** Yao Zhang, Jipeng Song, Yuzheng Lu, Meng Yi, Wancheng Lin, Mingtao Yao, Zhengning Luo, Genai Zhang, Lixiang Ding

**Affiliations:** Department of Spinal Surgery, Beijing Shijitan Hospital, Capital Medical University, No.10, Tieyi Road, Yangfangdian, Haidian District, Beijing, 10038 People’s Republic of China

**Keywords:** Iliac screw, Spinopelvic fixation, High grade spondylolisthesis, Spinal sagittal imbalance, Spino-pelvic parameter

## Abstract

**Background:**

Management of high-grade spondylolisthesis (HGS) remains challenging. Spinopelvic fixation such as iliac screw (IS) was developed to deal with HGS. However concerns regarding constructs prominence and increased infection-related revision surgery have complicated it’s use. We aim to introduce the modified iliac screw (IS) technique in treating high-grade L5/S1 spondylolisthesis and it’s clinical and radiological outcomes.

**Methods:**

Patients with L5/S1 HGS who underwent modified IS fixation were enrolled. Pre- and postsurgical upright full spine radiographs were obtained to analyze sagittal imbalance, spinopelvic parameters, pelvic incidence-lumbar lordosis mismatch (PI-LL), slip percentage, slip angle (SA), and lumbosacral angle (LSA). Visual analogue scale (VAS), Oswestry disability index (ODI) were evaluated pre- and postoperatively for clinical outcomes assessment. Estimated blood loss, operating time, perioperative complications and revision surgery were documented.

**Results:**

From Jan 2018 to March 2020, 32 patients (15 males) with mean age of 58.66 ± 7.77 years were included. The mean follow-up period was 49 months. The mean operation duration was 171.67 ± 36.66 min. At the last follow-up: (1) the VAS and ODI score were significantly improved (p < 0.05), (2) PI increased by an average of 4.3°, the slip percent, SA and LSA were significantly improved (p < 0.05), (3) four patients (16.7%) with global sagittal imbalance recovered a good sagittal alignment, PI-LL within ± 10° was observed in all patients. One patient experienced wound infection. One patient underwent a revision surgery due to pseudoarthrosis at L5/S1.

**Conclusion:**

The modified IS technique is safe and effective in treating L5/S1 HGS. Sparing use of offset connector could reduce hardware prominence, leading to lower wound infection rate and less revision surgery. The long-term clinical affection of increased PI value is unknown.

## Background

High-grade spondylolisthesis (HGS) is defined as greater than 50% slippage of a spinal vertebral body relative to an adjacent vertebral body as per Meyerding classification, and most often affects the alignment of the L5 and S1 vertebral bodies [1].

Surgery is indicated in HGS patients with back pain and/or radicular symptoms if conservative treatment fails [2]. Moreover, HGS invariably induce secondary changes in the regional pelvic anatomy and can thus produce global sagittal deformity, which might be another indication for surgery [3].

The surgical treatment of HGS remains challenging and is associated with significant controversies in terms of the optimal surgical technique [4–6]. Recently, partial reduction of HGS with the goal of slip angle reduction and lumbosacral kyphosis correction, combined with anterior column structural support, lead to greater stability at the lumbosacral junction, improved sagittal alignment and higher fusion rate [2, 7–10]. Since the advent of the Galveston technique to treat spinal scoliosis by Allen and Ferguson [11, 12], the concept and technique of spinal-pelvic fixation have been widely accepted and applied in diversity clinical practices. Achievement of high fusion rate has been reported with reliable security by iliac screw placement in spine disorders including HGS [13–15]. However, implant prominence at posterior superior iliac spine (PSIS) was the most unacceptable complication, which induced higher wound infection rate and increasing revision procedure due to surgical site infection [16]. Moreover, the usage of an additional offset-connector would complicate the procedure and prolong the operation duration. S2 alar iliac screw (S2AI) resolved the above-mentioned problems, but the high stress at sharp-angled junction of screw and shaft induce higher loosening rates in osteoporotic bone [17]. In addition, sacroiliac joint disturbance and spinopelvic parameters changing after fixation are deserve concerns [18, 19].

In this study, we introduced a modified unilateral iliac screw fixation technique which simplified the traditional procedure and decreased the hardware-related complications. We also reported the clinical and radiological outcomes, as well as the perioperative complications.

## Methods and materials

### Study design and patient selection

This was a prospective non-randomized study which was approved by the ethics committee of our institution (ethics committee of Beijing Shijitan hospital of Capital Medical University). All patients provided informed consent.

**Fig. 1:**
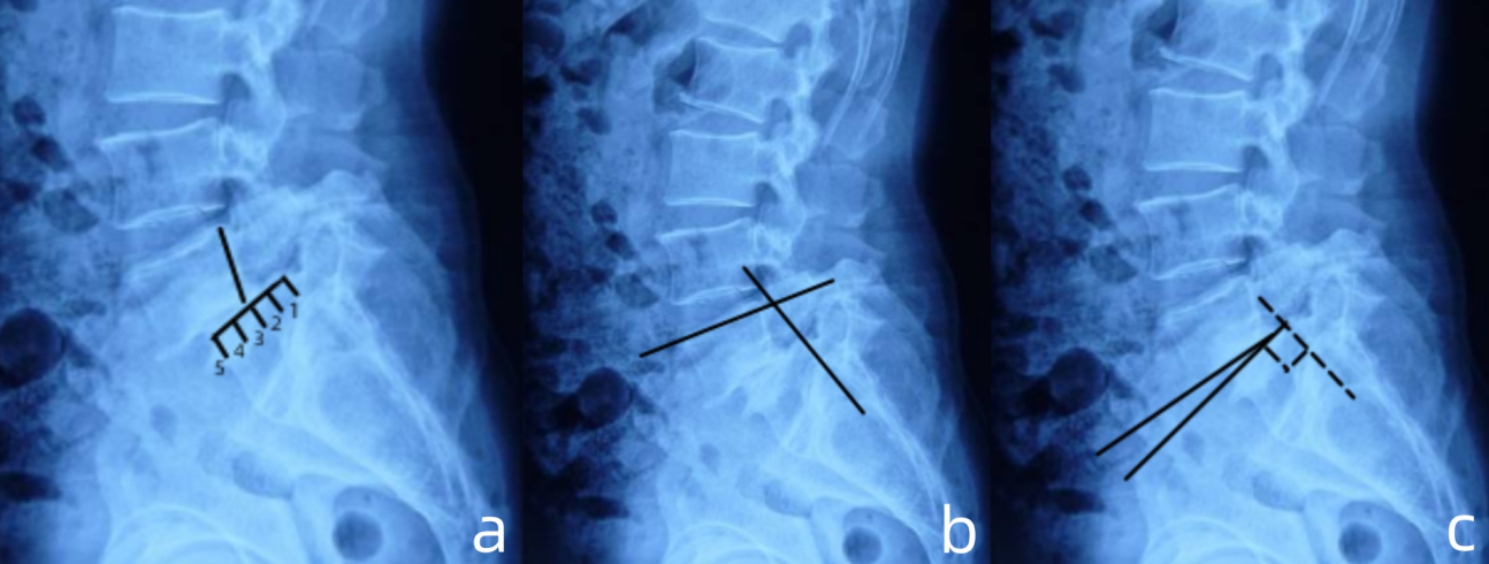
Schematic diagram of measurements of spondylolisthesis on lateral radiograph. (**a**). measurement of the transitional component of spondylolisthesis per Meyerding grade; (**b**). measurement of Dubousset’s lumbosacral angle; (**c**) measurement of Boxall’s slip angle

From January 2018 to March 2020, the patient who hospitalized in our spine center met the following criteria were enrolled in this study. Inclusion criteria: (1) age > 18 years old; (2) diagnose of high-grade L5/S1 spondylolisthesis (> 2 Meyerding grade); (3) severe low back pain or radiculopathy which was unresponsive to an over 3-month course of conservative treatment; (4) significant lumbosacral kyphotic deformity (measured by Dubousset’s lumbosacral angle and Boxall’s slip angle [Fig. 1a, b, c] ) causing unbalanced pelvic version (retroverted pelvic with high pelvic tilt/low sacral slope) and sagittal spinopelvic malalignment (the C7 plumb line fall in front of the femoral head and the offset between C7 plumb line and sacral vertical line > 4 mm). The exclusion criteria were: (1) previous spinal surgery history; (2) spinal diseases involving scoliosis, infection or malignant tumor; (3) poor general condition making the surgery impossible.

### Technique note

After general anesthesia, patient was positioned prone on the Allen table with the abdomen free-hanging to decrease bleeding and hip extension to recover lumbar lordosis. A midline incision was extended caudally to the spinous process of the lumbosacral junction. Within the same incision, the posterior superior iliac spine (PSIS) was palpated and was then exposed subperiosteally. The insertion point of IS was located 1 cm superomedial to the PSIS, which can be more suitable for the rod contouring. A small osseous recess (1 cm in diameter) at the entry point was made for screw head embeddedness. The trajectory was made at least 10 mm above the sciatic notch with an medial angle of 30° and a caudal angle of 25°-30°, pointing at the ipsilateral acetabulum. The caudal angle can be slightly reduced due to the lower insertion point, avoiding impingement of sciatic notch. Ball tip was used to confirm the intra-osseous trajectory, final position was identified by fluoroscope. The longest poly-axial pedicle screw for lumbar spine with 55 mm in length and 6.5 mm in diameter was used for IS fixation.

**Fig. 2:**
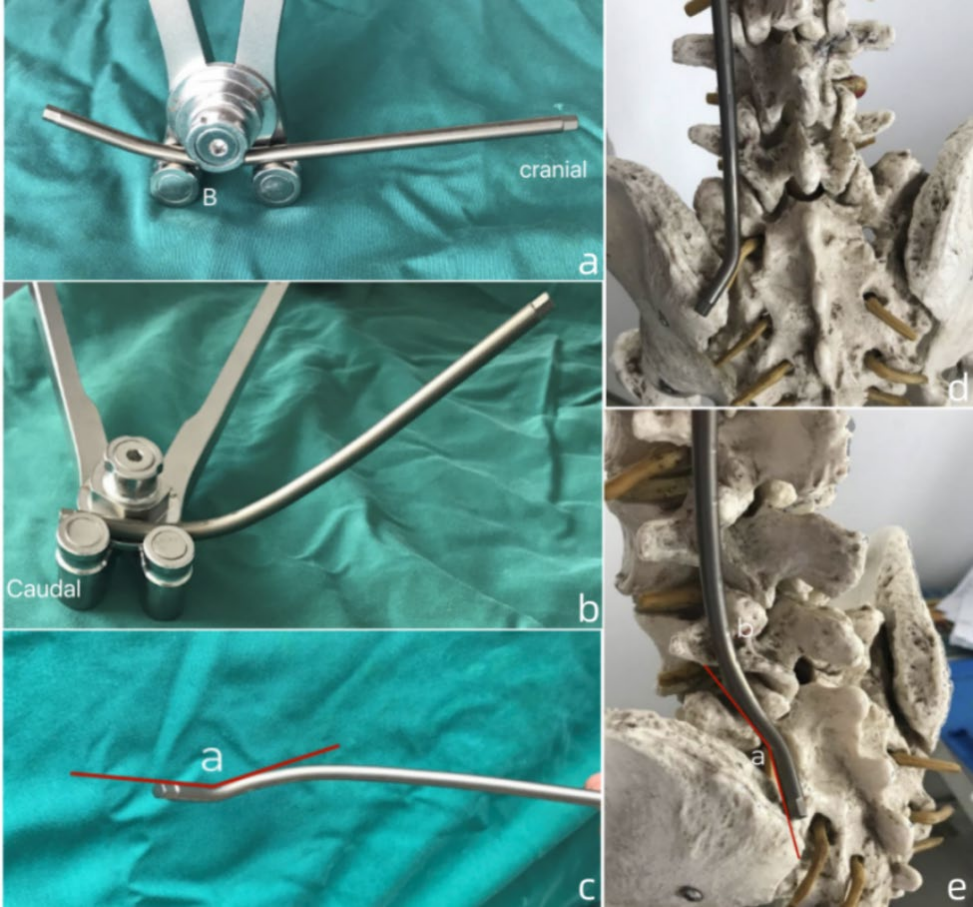
Illustration of rod contouring technique. (**a**). The first step: B stands for the bending point which is locates at the caudal 1/3 of the rod; (**b**), (**c**). the second step: angle “a” is formed by the distal part of rod and the sacral part of rod; (**d**), (**e**)s: the final step and position on a model, “a” indicates angle “a”

Rod contouring was the key procedure. First, the inferior 1/3 part of the the rod was contoured to fit the the lumbosacral curvature. Afterwards, an angulation of 150° between the distal end of rod and the sacral part of rod was made to facilitate the placement of S1 screw and IS [Fig. 2a, b, c, d, e].

Decompression procedure was performed by means of laminectomy and inferior facetectomy of the L5, as well as a laminotomy of S1 through a single posterior approach. The resected bones were morsellized for autograft. Partial reduction was completed through both cantilever effect and distraction technique between vertebral body and screw head of L5 and S1. The L5 nerve root was visually exposed and well-protected during the reduction procedure. Interbody autograft and anterior support with PEEK cage were performed in all patients. The levels that need to be decompressed and fused were dependent on individual situation.

### Postoperative management

Estimated blood loss, operating duration, postoperative complications and revision surgery were documented and evaluated. Patient were encouraged off-bed activity and gradual back muscle exercise one day after surgery. The duration of bracing was at least 3 months.

### Radiological assessment

**Fig. 3:**
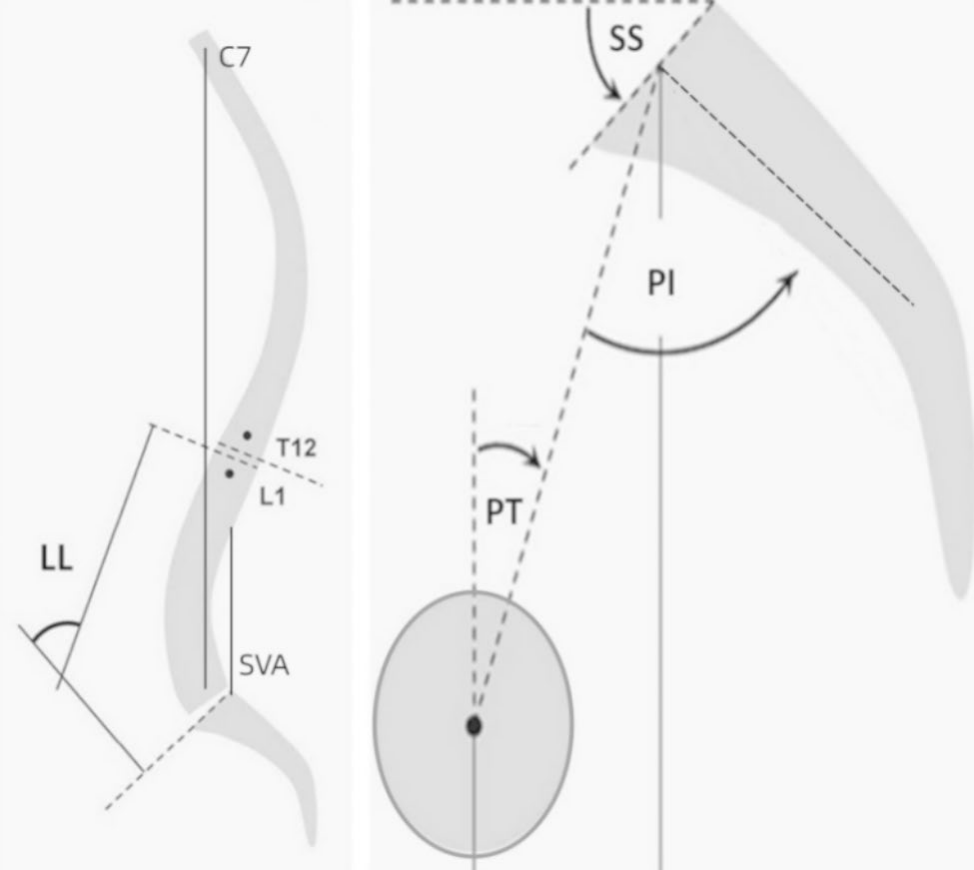
The measurement of sagittal spinopevlic parameters

Evaluation of radiographical parameters included slip grade, LSA, SA, pelvic incidence (PI), pelvic tilt (PT), sacral slope (SS), lumbar lordosis (LL), PI-LL mismatch, offset of C7PL - SVA [Fig. 3]. The above parameters were measured at the time of initial presentation and at the last follow-up by 2 independent senior surgeons in a blinded way and the averages were calculated for each parameter. The fusion status was initially evaluated on upright static radiographs by assessing the bony bridge between L5 and S1 vertebra. If the fusion status was not clear, computed tomography and/or dynamic radiographs of lumbar spine were then obtained for further evaluations.

### Evaluation of clinical outcomes

The clinical outcomes were assessed before surgery, at postoperative 6 months, postoperative 1 year, and at the last follow up. Visual Analog Scale (VAS) and Oswestry Disability Index (ODI) were used to assess back and/or leg pain, and functional capacity, respectively.

### Statistics

All data were represented as mean ± standard deviation (x ± s). The radiological parameters, VAS and ODI scores before and after surgery were compared using repeated-measures ANOVA. P < 0.05 was considered to be statistically significant. All analyses were conducted using SPSS 19.0 software (SPSS, Inc., Chicago, IL, USA).

## Results

**Fig. 4:**
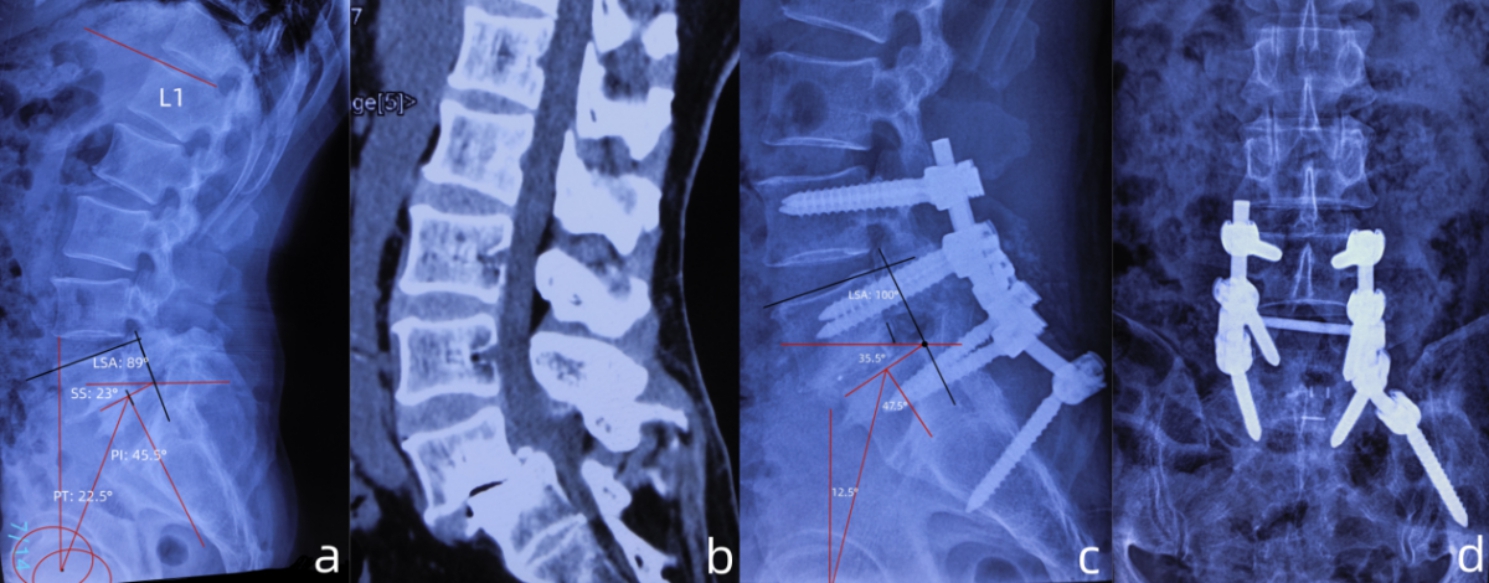
Case presentation of radiographic parameters measurements at pre- and post-operation. (**a**). lateral radiograph of a patient with grade 3 spondylolisthesis and retroverted pelvic (PT: 22.5°), the PI was 45.5°, the LSA was 89°, the PI-LL was +4.5°; (**b**). the sagittal CT image showed narrowing and “cleft-sign” of L5/S1 intervertebral space, as well as sclerosis of adjacent end-plates; (**c**). postoperative lateral radiograph showed reduction of slip and pelvic retroversion (PT: 12.5°), the PI was increased to 47.5°, the LSA was increased to 100°; (**d**). AP view of radiography showed satisfied position of the constructs

**Fig. 5:**
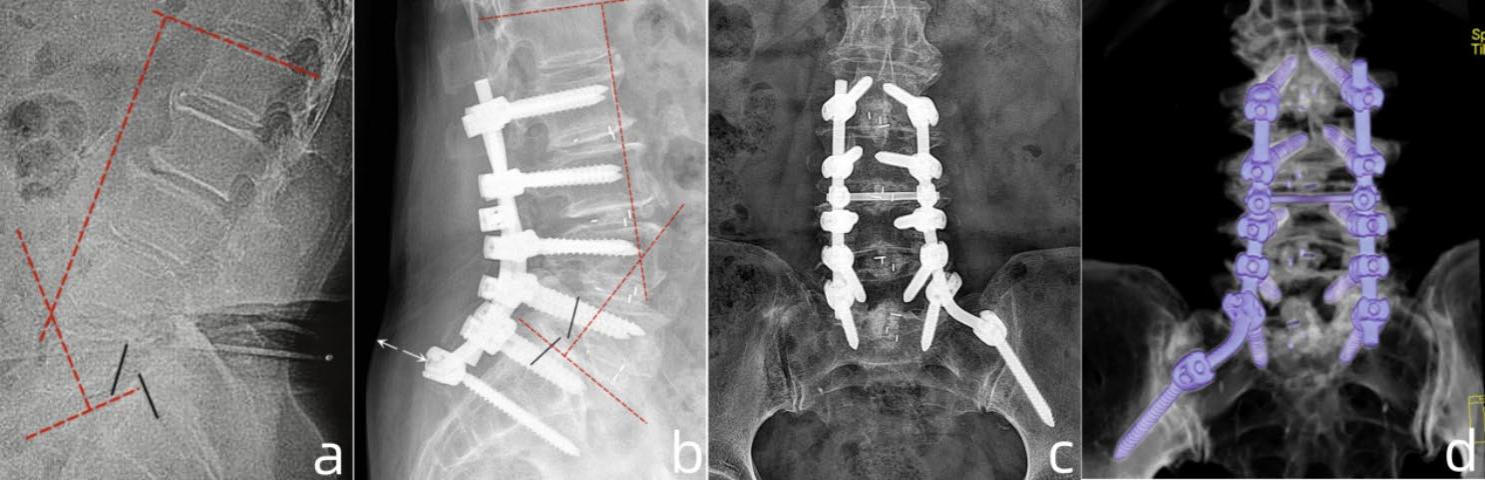
Case presentation of pre- and postoperative radiographic parameters measurements. (**a**). The lateral radiograph of a patient with grade 3 spondylolisthesis, the PI was 50°, the lumbar lordosis (LL) was 36°, PI-LL: +14°; (**b**), (**c**). postoperative radiograph showed the reduction of slip, the PI was 54°, the LL was 47° (PI-LL: +7°). Noting the construct was not prominent to the skin (white arrow in b); (**d**). postoperative 3-dimensional reconstruction on CT (posterior view) showed satisfied position of the constructs

A total of 32 (including 15 males) patients were enrolled, the mean follow-up period was 49 months, ranging from 13 to 55 months. The mean operation duration was 171.67 ± 36.66 (range: 120–240 min). The mean blood loss was 259.44 ± 89.53 ml (range: 160 to 520 ml) [Table 1]. All surgeries were successfully performed. The case illustration was showed in [Fig. 4] and [Fig. 5]. The VAS and ODI score were significantly improved at the final follow-up compared to those before operation (p < 0.05) [Table 2]. Slip percent improved by an average of 31.2% (p < 0.05). The improvement of SA and LSA were significant difference (p < 0.05). Four patients with sagittal imbalance (SVA > 4 mm) recovered a normal range (SVA < 4 mm) at the last follow-up. A PI-LL within ± 10° was observed in all patients at the last follow-up. At the last follow-up, the PI increased by an average of 4.3° [Table 3]. One patient (3.1%) experienced wound infection, the wound healing occurred 20 days after surgery through drape changing. One (3.1%) patient with rheumatoid arthritis and osteoporosis accepted a revision surgery due to pseudoarthrosis at L5/S1. One patient (3.1%) experienced L-5 radiculopothy immediately after surgery, the symptom improved 1 month after surgery with application of painkiller and mecobalamine.

**Table 1:** Baseline and surgical characteristics (N = 32)

**Age; range (years)**	58.66 ± 7.77; 47–77
**Gender**	
Male	15 (46.9%)
Female	17 (53.1%)
**Body Mass Index; range**	25.81 ± 2.15; 22–30
**Bone Mineral Density; range (T value)**	-1.05 ± 1.05; -3.5-0.8
**Smoking history**	4 (12.5%)
**Chronic comorbidity**	
hypertension	4 (12.5%)
diabetes mellitus	3 (9.4%)
rheumatoid arthritis	1 (3.1%)
osteoporosis	4 (12.5%)
**Fusion levels**	
2	18 (56.3%)
3	6 (18.7%)
4	5 (15.6%)
5	3 (9.4%)
**Mean operation duration and range (min)**	171.67 ± 36.66; 120–240
Mean operation duration and range according to different fusion levels (min)	
2	142.98 ± 19.98; 120–170
3	179.14 ± 24.23; 150–190
4	190.79 ± 43.14; 180–220
5	222.60 ± 10.32; 210–240
**Mean blood loss and range (ml)**	259.44 ± 89.53; 160–520
**Mean blood loss and range according to different fusion levels (ml)**	
2	221.87 ± 75.44; 160–310
3	293.69 ± 80.72; 210–350
4	389.80 ± 63.55; 330–450
5	467.44 ± 49.18; 410–520
**Postoperative complication**	
wound infection	1 (3.1%)
pseudoarthrosis	1 (3.1%)
radiculopathy	1 (3.1%)
**Revision surgery**	1 (3.1%)
**Total**	32 (100%)

**Table 2:** Clinical evaluation per follow-up interval

	Pre-op	Post 6-month	Post 1-year	Last-Fu	P^1^	P^2^	P^3^	P^4^
**VAS**								
back	7.33 ± 1.66	5.18 ± 2.01	3.25 ± 1.43	2.61 ± 1.33	**0.032**	**0.013**	**0.009**	0.169
leg	6.21 ± 1.10	3.71 ± 1.38	2.14 ± 0.51	1.62 ± 0.92	**0.028**	**0.015**	**0.012**	0.180
**ODI**	58.21 ± 10.38	15.43 ± 6.55	14.95 ± 5.46	10.39 ± 3.95	**0.036**	**0.027**	**0.021**	0.278

Pre-op: pre-operation; Post 6-month: 6 month post-operation; Post 1-year: 1 year post-operation; Last-Fu: last follow-up; P^1^: comparison between Post 6-month and Pre-op; P^2^: comparison between Post 1-year and Pre-op; P^3^: comparison between Last-follow up and Pre-op; P^4^: comparison between Last-Fu and Post 1-year; the bold indicate significant difference

**Table 3:** Radiological evaluation and comparison between preoperation and final follow up

	Pre-op	Last-Follow	P value
**PI**	50.25 ± 5.43	54.55 ± 4.84	P > 0.05
**PT**	21.08 ± 6.23	15.71 ± 5.44	**P < 0.05**
**SS**	29.17 ± 5.16	38.84 ± 6.06	**P < 0.05**
**C7PL-SVA (mm)**			
N1	10	3	
N2	8	2	
N3	7	2	
N4	6	1	
**LL**	40.50 ± 6.93	44.33 ± 5.28	P > 0.05
**PI-LL**	9.50 ± 3.55	6.21 ± 1.64	P > 0.05
**Slip percentage**	62.71 ± 5.51	31.54 ± 4.29	**P < 0.05**
**SLA**	76.77 ± 16.08	106.45 ± 8.13	**P < 0.05**
**SA**	27.92 ± 4.51	14.31 ± 5.18	**P < 0.05**

N1-4 indicate the serial number of patient with global spinal sagittal imbalance from 1 to 4; the bold indicates significant difference

## Discussion

With the tremendous advances in spinal instrumentation technique over the last quartercentury, reduction of HGS can now be accomplished more safely and effectively than ever before [2]. The primary rationale of reducing severe slip is to improve the global sagittal alignment through correcting the LSA, and consequently, improving the patient’s ability to stand upright.The additional advantage of a reduction procedure is improving intervertebral fusion through a increased bone-implant contact area [20–22]. Partial reduction of slip is proved to benot only effective, but safer than complete reduction fashion. First, HGS in adults have often reached a stable position, auto-fusion or ankylosis of the slipped level can occur, resulting in difficulties in anatomy reduction. Second, most of the total L5 nerve strain occurs during the second half of reduction [23]. Third, partial reduction can also lead to correction of slip angle which is related to the risk of progression and is the key to restoring sagittal alignment [10, 24]. Moreau et al. [25] treated 50 patients with HGS through a single posterior approach with IS fixation and partial reduction. The mean listhesis grade reduced by > 50%, the LSA was significantly improved. Seventeen patients (34%) showed postoperative radicular deficit. Hart et al. [26] treated 16 patients with modified Bohlman technique, slip percent reduced by 25%, one patient suffered L-5 radiculopathy. In these series of patients, slip percentage reduced by 31.2% (from average 62.71–31.54%). The SA and LSA significantly improved after surgery and remained stable at the last follow. One patient (4%) manifested right L5 nerve root palsy after surgery. In the premise of satisfied SA and SLA correction, proper less reduction of slip might have advantages of lower radicular injury rate.

Since Allen and Ferguson [27] introduced the landmark Galveston technique, the iliac screw fixation was then developed and was demonstrated to have superior biomechanical stability as compared to any of the preceding spinopelvic fixations [28]. However, wound complications, implant prominence and subsequent buttock pain are well-acknowledged drawbacks of IS technique [29, 30].

S2AI with deeper and medial insertion position, lower profile screw head and easier linkage with cephalad construct, was developed to be an alternative technique of traditional IS [30, 31]. S2AI is designed to purchase three-layer cortex. However, biomechanical study have shown that IS and S2AI constructs have demonstrated similar stability in terms of stiffness and load to failure [32, 33].

It is intriguing that the mode of implant failure may be different in IS and S2AI techniques. Shabtai et al. [34] demonstrated that a higher implant failure rate in the traditional IS group was a result from the disengagement of rod-iliac screw connector. The iliac screw and the side connector attachment creates a large moment arm with the proximal construct and a potential weak point in the construct. The implant failure etiology for S2AI was different from the traditional IS. Guler et al. [17] reported that S2AI harboured a higher risk of short-term acute failure in comparison of IS (35% in S2AI screws vs. 12% in traditional IS). They postulated that the acute angle developed between the screw head and shaft brought about a stress riser in the S2AI screw and caused it to fail when it crossed the sacral-iliac joint. According to Keorochana et al. [16], traditional IS screw fixation had higher postoperative complications and revisions than did S2AI fixation in adult and pediatric populations. Ishida et al. [35] demonstrated that the IS technique have higher rate of overall reoperation than the S2AI technique, but interestingly, the majority of reoperations in the IS group were attributable to surgical site infection. Similarly, Elder et al. [36] also concluded that the higher rates of revision surgery was caused by iliac screw head being prominent in the subcutaneous tissue, causing symptoms such as hip or buttock pain, may in turn lead to wound complications breakdown and infection.

We modify the traditional IS technique in a simplified fashion to deal with the drawbacks mentioned above. First, we use the lumbar pedical screw with maximum size (6.5 mm in diameter, 55 mm in length), which is smaller than the traditional IS (8 mm in diameter and 80-100 mm in length), thus we can choose a more inferior and medial insertion point to avoid hardware prominence. Additionally, due to the screw head is smaller than that of traditional IS, the embeddedness of screw head is deeper and in turn increase 1cm of effective length. Second, we use the traditional rod instead of using a off-set connector, with easy rod contouring technique, to simplify the procedure. The rod contour between S1 and IS screw is smooth and obtuse-angulate, which mimics the physical curvature, and is able to maintain the original rod rigidity. Moreover, a short rod between S1 pedical screw and IS is able to reduce the moment arm and to provide enough constructive stability. In the present study, one patient (4.1%) suffered wound infection, which is lower than the previous reports of 4.2–20% [37–39]. The VAS and ODI scores were significantly improved at the last follow-up, no patient suffered severe hip or buttock pain.

We performed unilateral IS fixation in all patients. There are several studies that comparing the outcomes between bilateral and unilateral IS fixation. Tomlinson, et al. [40] performed a biomechanical study on sixteen porcine spines to compared the difference of mechanical stiffness between unilateral and bilateral IS fixation. In this study, the spines were instrumented with pedicle screws and 5.5-mm titanium rods from L1 to S1, the ilium were instrumented with IS bilaterally or unilaterally. They concluded that no biomechanical differences between bilateral and unilateral iliac screw fixation were found. The instrumentation fashion in Tomlinson’s study is similar to the that of human spine and ilium, which makes the conclusion convincing. Saigal et al. [41] retrospectively studied 102 patients underwent spinal fixation extended to pelvis. They compared the differences of postoperative complications including reoperation, L5–S1 pseudarthrosis, sacral insufficiency fracture, hardware prominence, iliac screw loosening, and infection between patients with uni- and bilateral IS fixation. They found that single versus dual IS fixation led to comparable complication rates, and inserting bilateral IS (vs. unilateral) produced no added clinical benefit in most cases. In these series of patients, the fusion rate was 95.8%. One patient with rheumatoid arthritis and severe osteoporosis was diagnosed to be fusion failure and underwent the revision surgery. Three patients (12.5%) manifested asymptomatic IS loosening after fusion has occurred. The outcomes proved that the modified unilateral IS fixation was able to provide enough fixation stability.

Historically, PI was considered a constant anatomical parameter after maturity in the absence of pelvic fractures or sacropelvic tumor resection [42, 43]. Actually, the motion of sacroiliac joint (SIJ) which was affected by sitting, supine position or others locomotion activities, would cause the change of PI. The magnitude of SIJ movement in adulthood has been reported ranging from 1 to 4° of rotation [44]. Additionally, the laxity of SIJ due to degeneration is considered to be the fundamental of PI change [45]. According to Dreyfuss et al. [46], SIJ has less resistance to rotation force during the motion. Thus, we postulate that: on the one hand, the patient positioning of slight hip extension during surgery might cause the anterior rotation of the sacrum, resulting in intraoperative increase of PI, which would be finally maintained by the application of sacroiliac fixation; on the other hand, incremental stress to SIJ after spinopelvic fixation accelerates SIJ degeneration, inducing an increasing motion. In this study, we find the PI is increased by an average of 4.3° at the final follow-up, which is smaller than the findings of Lee et al. (5.9°) [47] and larger than the results of Ishida et al. (2.9°) [35]. The dramatically decrease of PI after S2AI fixation was well-introduced by many authors [19, 35, 47, 48]. The change in PI after S2AI instrumentation may be due to direct fixation and modification of the SIJ, which have been reported by cadaveric study [49]. From our point of view, the IS would have advantage over S2AI regarding the PI change. An obvious decreased PI value may potentially affect the self-adjust ability of sagittal balance at spinopelvic area, as the SIJ is the last mobilizable joint after lumbosacral fusion. Moreover, a pelvic with larger PI would have greater compensation ability than the pelvic with small PI [50]. However, postoperative changes in spinopelvic parameters are a complicated phenomenon. The mechanism of how spinal alignment may be affected by instrumentation and how it is translated into clinical outcomes is still unknown, further study is needed.

There are several limitations in this study. First, the sample size was small, involving only 32 patients. Larger sample sizes will be needed to provide stronger evidence to our conclusions. Second, the follow-up period was short with an average of 49 months. Prolonged follow-up was needed to figure out the mechanism of PI change and the consequent long-term outcomes. Third, we performed unilateral IS fixation in all patients. The clinical and radiological outcomes by now proved that the constructs was able to provide enough fixation strength for reduction and stability, however biomechanical study would be of great necessity.

## Conclusion

The modified IS technique is safe and effective in treating L5/S1 HGS with reliable constructive stability, good ability for slip reduction and satisfactory fusion rate. It is easy to apply through designed rod-contouring technique. Sparing use of offset connector could simplify the complexity of surgery and avoid hardware prominence, which lead to a lower wound infection rate and less infection-related revision surgery. The clinical affection of increased PI value is still unknown, future study with larger sample size and longer follow up period is needed.

## Data Availability

The data analyzed during the current study are available from the corresponding author on reasonable request.
